# Supplementary material for: Genetic reversal of the globin switch concurrently modulates both fetal and sickle hemoglobin and reduces red cell sickling
Source: Nat Commun. 2023 Sep 20;14:5850. doi: 10.1038/s41467-023-40923-5 (PMC10511721; doi:10.1038/s41467-023-40923-5)
Supplement: Supplementary file 1 — Supplementary Information [file 41467_2023_40923_MOESM1_ESM.pdf]

Genetic reversal of the globin switch concurrently modulates both fetal and sickle hemoglobin and reduces red cell sickling

Authors: Daniel C. De Souza[*1,2,3], Nicolas Hebert[*4,5,6], Erica B. Esrick[7], M. Felicia Ciuculescu[8], Natasha M. Archer[7,8,9], Myriam Armant[8], Étienne Audureau[10,11], Christian Brendel[7,9], Giuseppe Di Caprio[1,9,12], Frédéric Galactéros[5,6], Donghui Liu[8], Amanda McCabe[8], Emily Morris[8], Ethan Schonbrun[1], Dillon Williams[13], David K. Wood[13], David A. Williams[+7,8,9*], Pablo Bartolucci[+5,6*], John M. Higgins[+1,2,3*]

Affiliations: [1]Center for Systems Biology, Massachusetts General Hospital, Boston, MA, [2]Department of Systems Biology, Harvard Medical School, Boston, MA, [3]Department of Pathology, Massachusetts General Hospital, Boston, MA, [4]French Blood Establishment (EFS), Créteil, France, [5]Paris-East Créteil University, IMRB, Laboratory of excellence LABEX, Créteil, France [6]Paris-East Créteil University, Henri Mondor University Hospitals, APHP, Sickle cell referral center – UMGGR, Créteil, France, [7]Dana-Farber/Boston Children's Cancer and Blood Disorders Center, Boston, MA, [8]Boston Children's Hospital, Harvard Medical School, Boston, MA, [9]Department of Pediatrics, Harvard Medical School, Boston, MA, [10]INSERM U955 Team CEpiA, Paris-East Créteil University, Créteil, France, [11]Department of Public Health, Henri Mondor University Hospitals, APHP, Créteil, France, [12]Program in Cellular and Molecular Medicine, Boston Children's Hospital, Boston, MA, [13]Department of Biomedical Engineering, University of Minnesota, Minneapolis, MN

Corresponding authors: David A. Williams (david.williams2@childrens.harvard.edu), Pablo Bartolucci (pablo.bartolucci@aphp.fr), and John M. Higgins (higgins.john@mgh.harvard.edu)

SUPPLEMENTARY INFORMATION

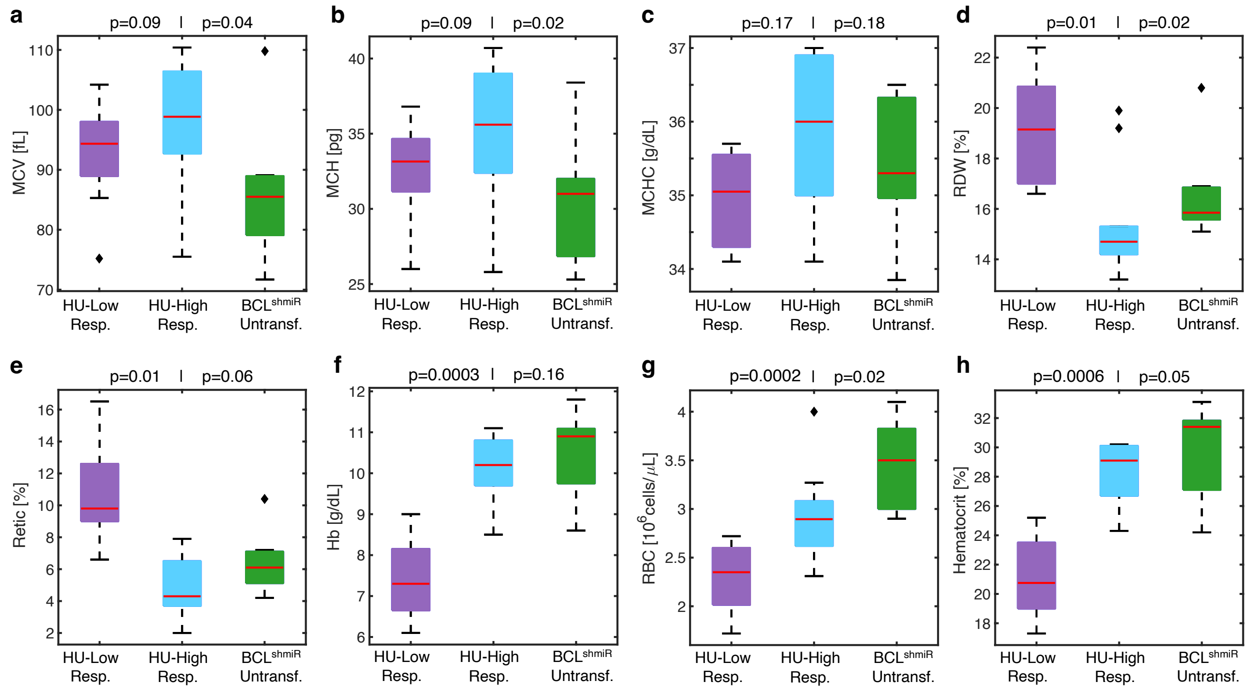

Figure S1 **Hematologic parameters for all 7 untransfused BCL^{shmiR}-patients and all 18 patients from the HU High\Low Responder cohorts.** On top of each panel the p-value in the left side compares median of Untransfused BCL^{shmiR}-timepoints after 4.5 months (n = 7; green boxes) with HU Low Responders cohort (n = 8; purple boxes) while the p-value in the right side compares Untransfused BCL^{shmiR} with HU High Responders values (n = 10; blue boxes). MCV (**a**), MCH (**b**), RDW (**d**), and RBC levels (**g**) showed a significant difference for Untransfused BCL^{shmiR} vs. HU High Responders cohort, while the differences in their MCHC (**c**), Reticulocyte fraction (**e**), Hemoglobin (**f**), and Hematocrit levels (**h**) did not reach statistical significance. Boxplot properties and the method used to compute the p-values in this study are described in **Methods, Data analysis, statistics, and reproducibility**. Source data are provided as a Source Data file.

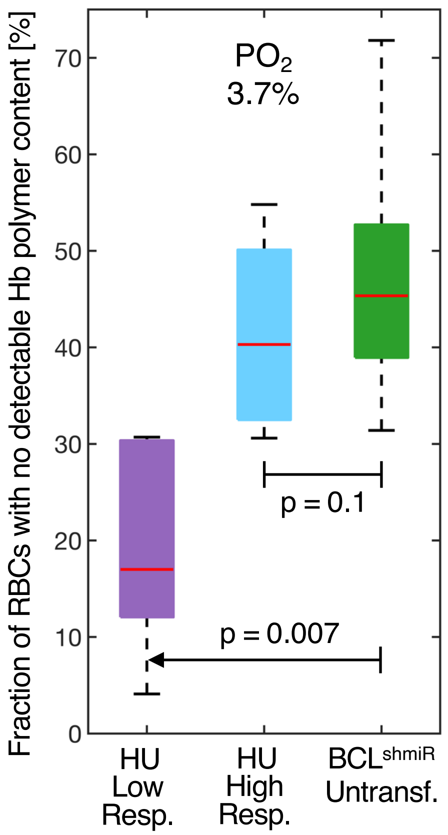

Figure S2 **Fractions of RBCs with no detectable Hb polymer content after BCL11A inhibition or HU treatment.** The percentage of RBCs that have no detectable Hb polymer content at 3.7% oxygen tension was measured in vitro in Untransfused BCL^{shmiR} (n = 7; green boxes), in HU High Responders (n = 10; blue boxes), and in HU Low Responders (n = 8; purple boxes). Boxplot properties and the method used to compute the p-values in this study are described in **Methods, Data analysis, statistics, and reproducibility**. Source data are provided as a Source Data file.

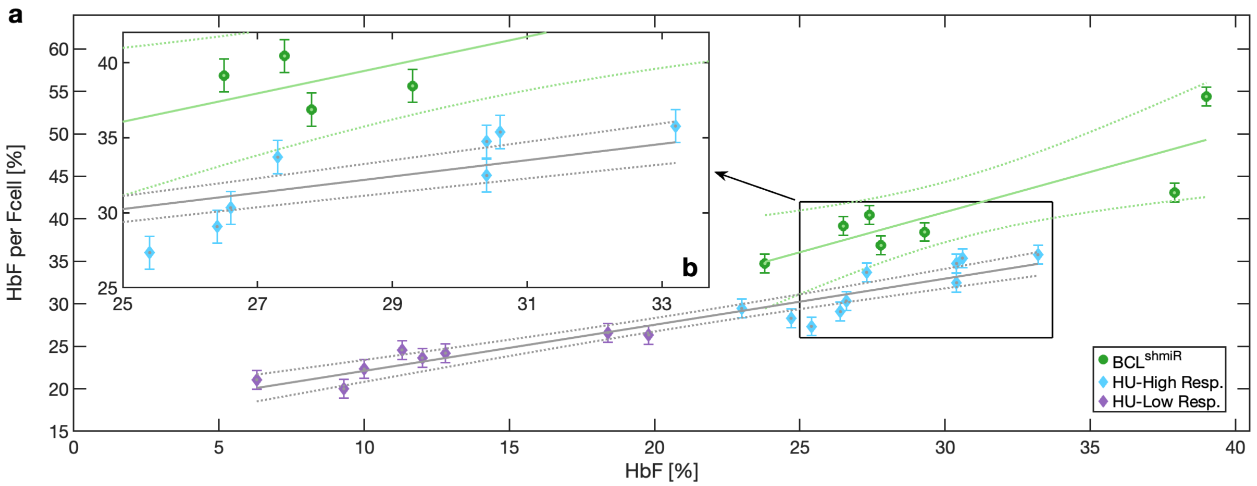

Figure S3 **BCL11A inhibition induces higher levels of HbF content per F-cell compared to HU according to total blood HbF%.** Panel **a** shows HbF expressed as the percentage of total HbF per F-cell according to whole blood HbF percentages for Untransfused BCL^{shmiR} (n = 7; green circles), HU High

Responders (n = 10; blue diamonds), and HU Low Responders (n = 8; purple diamonds). The percentages of total HbF per F-cell are estimated as the ratio of total HbF% to F-cell% and assume that the HbF content in the non F-cells is negligible. Green solid line shows linear regression for Untransfused BCL^{shmiR} points while the gray solid line indicates the linear regression for HU High Responders plus HU Low Responders data points. Dashed lines show the 95% CI for the correspondent cohorts. For each data point the bottom and upper bar indicates a 1.1% measurement uncertainty. Inset **b** shows a range of HbF levels where Untransfused BCL^{shmiR} and HU High Responders can be distinguished within the 95% CI. Source data are provided as a Source Data file.

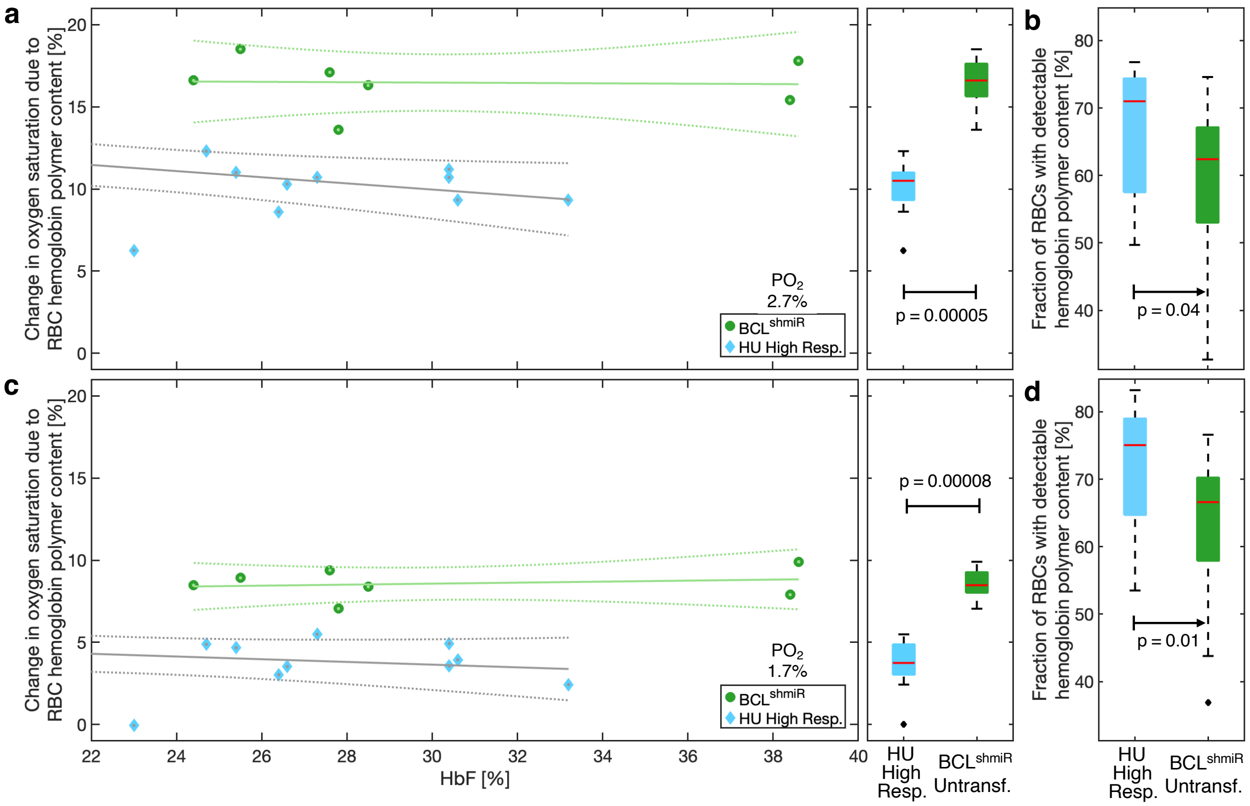

Figure S4 **BCL11A inhibition is associated with (i) more RBCs protected from polymerization and (ii) greater relative protection from polymerization per treatment-responsive RBC at lower physiologic oxygen tensions compared to HU across the range of total blood HbF% in these cohorts.** Panels **a** and **c** show the difference in oxygen saturation levels between the average treatment-responsive RBC (with no detectable Hb polymer content) and the average unprotected RBC whose oxygen saturation is reduced by Hb polymerization, at 2.7% and 1.7% oxygen tensions. Vertical axis shows the difference in oxygen saturation in RBCs for Untransfused BCL^{shmiR} (n = 7) and HU High Responders (n = 10). The green solid line and dashed lines show linear regression and 95% CI for BCL^{shmiR} data points while the gray solid line and dashed lines indicate the linear regression and 95% CI for HU High Responders and HU Low Responders data points (not shown here, data points are available in Table S1). Boxplots comparing both cohorts at 2.7% and 1.7% oxygen tensions are shown on the right. Panels **b** and **d** compare the fraction of RBCs with detectable hemoglobin polymer content for both cohorts at 2.7% and 1.7% oxygen tensions and show that BCL^{shmiR} is associated with fewer RBCs unprotected from polymerization at lower physiologic oxygen tension. Boxplot properties and the method used to compute the p-values in this study are described in **Methods, Data analysis, statistics, and reproducibility**. Source data are provided as a Source Data file.

Table S1. Individual Data.*

Cohort**	Patient	Age	Time Since Infusion/HU Treatment	RBCs ≥10pg Fig1b	RBCs >70% Fig1c	RBCs 1.7% Fig2c	RBCs 2.7% Fig2d	HbF Fig2e	F-cell Fig2f	HbF/ F-cell Fig3a	ΔHbS Fig4d§	MCV FigS1a	MCH FigS1b	MCHC FigS1c	RDW FigS1d	Retic FigS1e	Hb FigS1f	RBC FigS1g	HCT FigS1h	RBCs 3.7% FigS2	RBCs Oxy Sat FigS4a	RBCs Oxy Sat FigS4c	RBCs 3.7% FigS2	HbF¶	F-cell¶	HbF per Fcell‡	HbS per Fcell±	WBC
		years	months	%	%	%	%	%	%	%	-	fL	pg	g/dL	%	%	g/dL	$10^6 g/\mu L$	%	%	%	%	%	%	%	pg	pg	$10^3 cells/\mu L$
BCL-U	2	21	48	49.8	51.4	29.3	34.3	23.3	67.5	34.9	-0.76	89.1	31.3	35.0	15.9	6.1	10.9	3.5	31.4	43.3	16.6	8.5	43.3	24.2	70.2	10.9	20.3	11.2
BCL-T	3	26	38	46.1	-	22.9	26.8	23.4	62.7	36.8	-	89.2	30.0	32.9	17.0	8.7	9.9	3.4	30.4	30.9	-	-	30.9	24.0	64.3	11.3	18.7	5.4
BCL-U	4	24	39	38.5	41.6	35.3	37.6	28.9	75.4	38.2	-0.73	85.5	31.0	36.4	16.9	6.8	11.1	3.6	30.7	47.5	16.3	8.4	47.5	30.2	79.1	11.9	19.0	7.5
BCL-U	6†	16	35	55.7	63.3	47.0	48.5	38.7	71.9	53.4	-0.83	77.8	25.8	33.9	20.8	5.5	10.6	4.1	31.6	58.5	17.8	9.9	58.5	40.7	75.6	13.9	12.1	7.8
BCL-U	7	12	31	52.0	57.3	39.4	46.9	25.5	64.0	39.7	-0.73	83.0	30.0	36.1	16.7	4.2	11.8	3.9	33.1	49.8	18.5	8.9	49.8	26.8	67.4	11.9	18.1	5.7
BCL-U	8	7	26	50.1	46.9	41.4	39.8	37.9	86.1	43.1	-0.74	88.5	32.3	36.5	15.1	7.2	9.4	2.9	25.9	45.0	15.4	7.9	45.0	39.0	90.0	14.0	18.5	8.0
BCL-U	9	12	21	44.0	37.6	27.5	30.5	27.9	75.4	36.9	-0.49	109.8	38.4	35.0	15.5	10.4	11.1	2.9	31.9	34.9	13.6	7.1	34.9	28.7	77.8	14.3	23.7	6.9
BCL-T	10	20	22	40.1	-	14.2	18.0	11.1	37.3	27.8	-	82.7	28.8	35.0	18.6	6.1	7.6	2.6	21.7	24.8	-	-	24.8	11.7	38.8	8.1	20.9	7.0
BCL-U	11	22	16	54.7	64.6	42.2	43.7	27.6	68.6	39.7	-0.67	71.7	25.3	35.3	15.8	5.0	8.6	3.3	24.2	48.6	17.1	9.4	48.6	28.8	72.5	10.0	15.1	5.6
HU-H	H1	5	46	24.4	21.1	24.6	28.7	23.0	78.1	29.4	-0.12	92.7	32.4	35.0	19.9	7.8	8.5	2.6	24.3	50.1	6.3	-	50.1	24.3	82.6	9.5	22.9	14.7
HU-H	H2	2	11	43.2	37.4	19.8	25.6	24.7	87.3	28.3	-0.13	84.1	29.7	35.3	14.6	7.9	9.7	3.3	27.5	33.0	12.3	4.9	33.0	26.3	92.9	8.4	21.3	11.5
HU-H	H3	9	62	56.5	49.0	25.3	29.3	25.4	92.9	27.3	-0.42	96.4	33.2	34.5	14.7	6.5	10.1	3.0	29.3	42.6	11.0	4.7	42.6	26.8	98.2	9.1	24.1	6.4
HU-H	H4	5	46	32.0	26.9	27.8	31.0	26.4	90.8	29.1	-0.27	100.0	35.8	35.8	14.7	4.5	10.8	3.0	30.2	38.0	8.6	4.7	38.0	28.4	97.5	10.4	25.4	9.9
HU-H	H5	16	108	42.3	31.4	16.8	23.2	26.6	87.7	30.3	-	108.3	40.1	37.0	14.8	5.4	11.1	2.8	30.0	30.6	10.3	3.0	30.6	28.4	93.5	12.2	27.9	6.6
HU-H	H6	13	61	43.4	38.2	38.5	42.5	27.3	81.0	33.7	-0.51	110.4	40.7	36.9	14.2	4.1	9.4	2.3	25.5	46.7	10.7	5.5	46.7	28.7	85.1	13.7	27.0	5.8
HU-H	H7	12	44	58.8	50.0	21.0	24.6	30.4	93.6	32.5	-0.29	105.3	38.6	37.0	13.6	4.1	10.7	2.8	28.9	32.5	10.7	4.9	32.5	32.4	99.9	12.5	26.1	6.0
HU-H	H8	2	10	26.5	23.8	46.5	50.3	30.4	87.5	34.7	-0.34	75.5	25.8	34.1	19.2	2.0	10.3	4.0	30.2	53.3	11.2	3.6	53.3	32.3	92.9	9.0	16.8	5.7
HU-H	H9	3	38	43.9	40.1	22.5	26.0	30.6	86.5	35.4	-0.35	97.7	35.4	36.2	13.2	3.7	10.9	3.1	30.1	32.2	9.3	3.9	32.2	32.6	92.1	12.5	22.9	7.2
HU-H	H10	10	156	51.1	40.9	35.3	46.6	33.2	92.8	35.8	-	106.4	39.0	36.7	15.3	3.2	9.8	2.5	26.7	54.8	9.3	2.4	54.8	35.5	99.1	14.0	25.0	6.4
HU-L	L1	23	120	27.4	43.6	4.0	5.6	6.3	29.9	21.1	-	75.2	26.0	34.5	20.7	9.1	6.7	2.6	19.4	19.0	-	-	19.0	6.8	32.4	5.5	20.5	6.4
HU-L	L2	13	144	16.1	32.3	2.9	3.7	9.3	46.5	20.0	-	100.6	35.5	35.3	22.4	16.5	6.1	1.7	17.3	11.7	-	-	11.7	9.8	48.9	7.1	28.4	15.9
HU-L	L3	14	86	21.2	19.9	5.6	7.8	10.0	44.8	22.3	0.018	104.2	36.8	34.1	21.0	10.0	7.8	2.1	22.1	15.0	9.7	2.1	15.0	10.5	47.3	8.2	28.6	8.5
HU-L	L4	8	24	26.5	24.3	10.8	12.6	11.3	46.0	24.6	-0.20	93.3	33.2	35.6	16.8	10.2	6.9	2.1	19.4	12.4	15.7	8.1	12.4	11.9	48.5	8.2	25.0	10.9
HU-L	L5	4	59	6.3	5.5	49.3	62.7	12.0	50.8	23.6	0.15	85.3	29.7	34.8	18.7	6.6	7.7	2.6	22.1	69.3	16.4	8.0	69.3	12.9	54.6	7.0	22.7	12.7
HU-L	L6	9	73	22.1	21.9	1.6	2.7	12.8	52.9	24.2	-0.19	95.4	33.8	35.5	19.6	15.0	6.6	2.0	18.6	4.1	11.3	2.9	4.1	13.5	55.7	8.2	25.6	10.7
HU-L	L7	4	43	15.9	12.7	15.6	21.9	18.4	69.2	26.6	-0.04	92.6	33.1	35.7	16.6	9.6	9.0	2.7	25.2	30.7	14.4	5.1	30.7	19.8	74.6	8.8	24.3	10.8
HU-L	L8	3	32	36.5	32.5	19.5	27.1	19.8	75.2	26.3	-0.23	95.4	32.6	34.1	17.2	8.9	8.5	2.6	24.9	30.0	14.2	5.4	30.0	21.3	80.8	8.6	24.0	6.7
BCL-U	Median	16.0	31.0	50.1	51.4	39.4	39.8	27.9	71.9	39.7	-0.7	85.5	31.0	35.3	15.9	6.1	10.9	3.5	31.4	47.5	16.6	8.5	47.5	28.8	75.6	11.9	18.5	7.5
HU-H	Median	7.0	46.0	43.3	37.8	25.0	29.0	27.0	87.6	31.4	-0.3	98.9	35.6	36.0	14.7	4.3	10.2	2.9	29.1	40.3	10.5	4.7	40.3	28.5	93.2	11.3	24.6	6.5
HU-L	Median	8.5	66.0	21.7	23.1	8.2	10.2	11.7	48.7	23.9	-0.1	94.4	33.2	35.1	19.2	9.8	7.3	2.4	20.8	17.0	14.3	5.2	17.0	12.4	51.8	8.2	24.7	10.8

* For each BCL^{shmiR} subject the data represents median values for data >= 5 months after treatment. Source data are provided as a Source Data file.

** BCL-U means Untransfused BCL^{shmiR}, BCL-T means Transfused BCL^{shmiR} , HU-H means HU High Responders, HU-L means HU Low Responders.

§ In Figure 4d we also measured ΔHbS for the 5 individuals from the BCL ^{shmiR} Baseline cohort: -0.0128, 0.0964, -0.0671, 0.0158, -0.0428.

¶ HbF anf Fcell values are presented as HbF/(HbF + HbS) and Fcell/(HbF + HbS).

† HbS/Beta Zero Thalassemia.

‡ The average HbF per F-cell was calculated with the following formula: MCH × HbF/Fcell. This estimate assumes that the Non-Fcells have a negligibe HbF content, i.e., the average HbS content per Non-Fcell is equal to the MCH.

± The average HbS per F-cell was calculated with the following formula: MCH - MCH × HbF/Fcell. As the average HbF per Fcell, this estimate assumes that the Non-Fcells have a negligibe HbF content.

Table S2. Vector Copy Number of Peripheral Blood and Bone Marrow (Copies per Diploid Genome)*									
Source and Time Point	Patient 2	Patient 3	Patient 4	Patient 6	Patient 7	Patient 8	Patient 9	Patient 10	Patient 11
Peripheral Blood									
6 months									
Whole Blood	0.42	1.49	0.61	1.19	1.04	0.69	0.50	0.23	0.86
CD19+	0.50	1.21	0.50	1.13	0.66	0.64	0.51	0.22	0.78
GlyA+, CD71+	0.20	2.85	0.96	1.35	0.76	0.68	0.76	0.21	1.20
Bone Marrow									
6 months									
CD34+	0.53	1.52	0.60	1.20	0.86	0.59	0.58	0.25	0.97
CD15+	0.65	1.59	0.79	1.65	1.25	0.77	0.52	0.20	0.79
GlyA+, CD71+	0.63	1.62	0.80	1.42	1.34	0.73	0.73	0.27	1.04

* Data from patients 2, 3, 4, 6, 7, and 8 are reproduced from Esrick et al. 2021[22]. Source data are provided as a Source Data file.

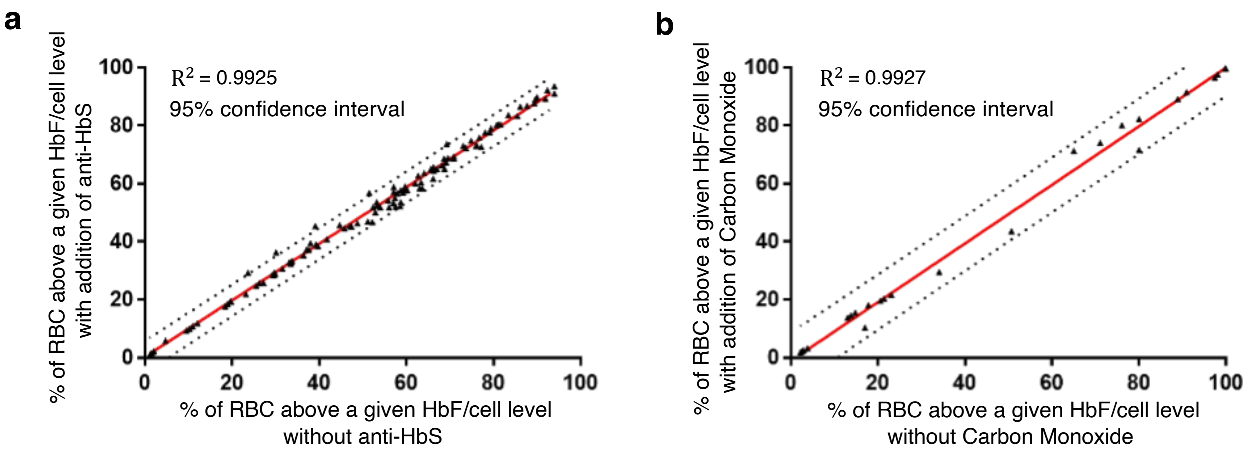

Figure S5 **Control experiments show that anti-HbS and the presence of polymer do not significantly interfere with the HbF measurement.** Experiments were conducted separately on the same samples with or without anti-HbS fluorescent antibody (**a**) and with or without carbon monoxide which impedes HbS polymerization (**b**). Data represents the fractions of RBCs with more than a threshold level of HbF/RBC (namely the percent of cells containing at least 2, 4, 6, 8 and 10 pg of HbF) using n=7 BCL^{shmiR} (total of 22 time points during their follow-up) and n=5 SS patients with HbF ranging from 0.7 to 14.5%, for addition of anti-HbS and carbon monoxide, respectively. Dashed lines represent 95% confidence intervals. $R^2 > 0.99$ in both cases. Source data are provided as a Source Data file.

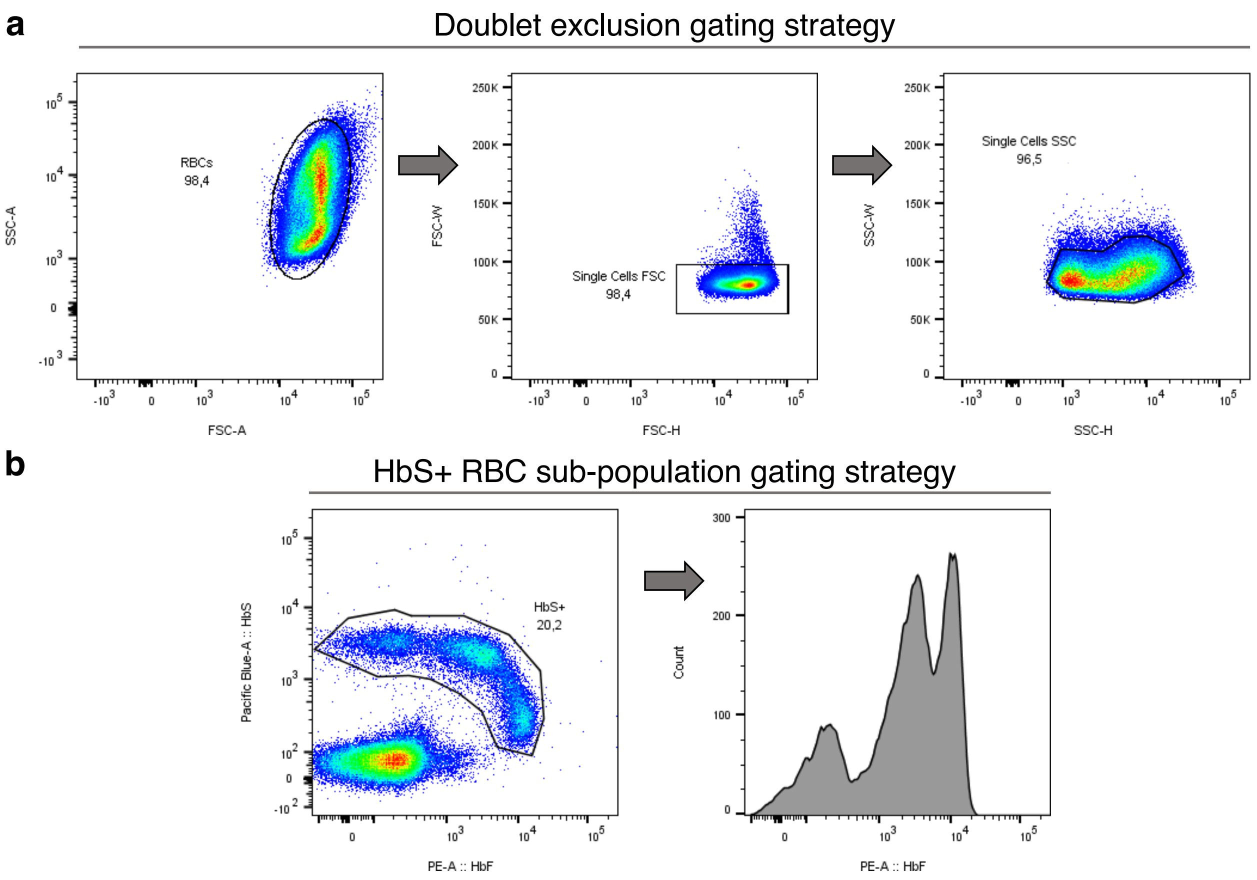

Figure S6 **Representative FACS dot plots and histogram from patient BCL-009 at M2 illustrating the gating strategy applied. (a)** Red blood cells were first gated on a forward scatter area (FCS-A) versus side scatter area (SSC-A) plot. Doublet exclusion was performed by selecting single cells based on FSC-H (FSC-Height) plotted versus FSC-W (FSC-Width), and then based on SSC-H versus SSC-W. **(b)** In the case of HbF quantification in only HbS-positive cells, the latter population was gated on HbS-PB-A plotted versus HbF-PE-A. Source data are provided as a Source Data file.

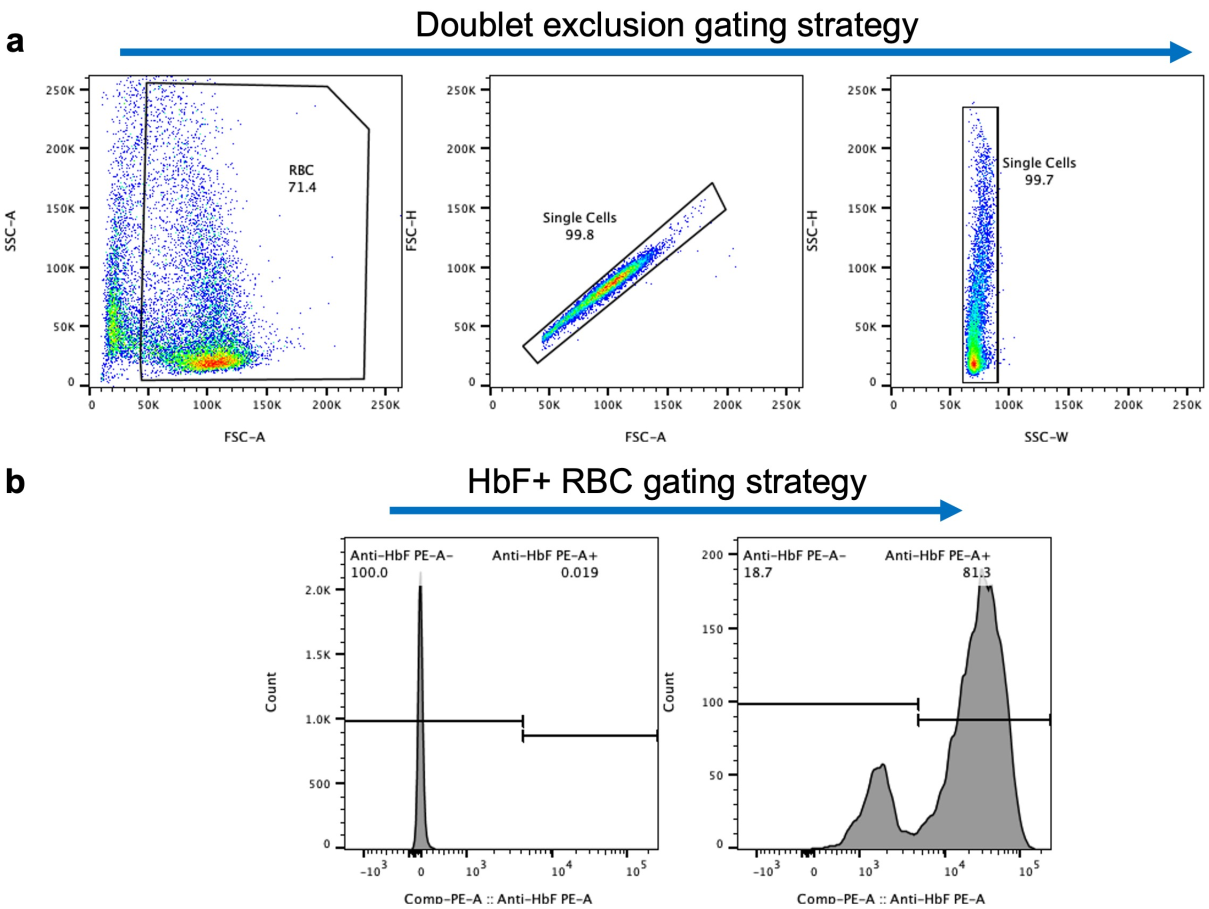

Figure S7 **Representative FACS dot plots and histograms from BCL-008 patient at M24 post GT illustrating the F-cell gating strategy. (a)** RBCs were gated on an FCS-A versus SSC-A plot, followed by doublets exclusion as described in Figure S6**a**. **(b)** Representative histograms are shown for a BCL-008 unstained sample (left panel) and a BCL-008 HbF-PE-stained sample (right panel). Source data are provided as a Source Data file.
