# Supplementary material for: Genetic reversal of the globin switch concurrently modulates both fetal and sickle hemoglobin and reduces red cell sickling
Source: Nat Commun. 2023 Sep 20;14:5850. doi: 10.1038/s41467-023-40923-5 (PMC10511721; doi:10.1038/s41467-023-40923-5)
Supplement: Supplementary file 3 — Reporting Summary [file 41467_2023_40923_MOESM3_ESM.pdf]

nature portfolio

Corresponding author(s): David A. Williams, Pablo Bartolucci, and John M. Higgins

Last updated by author(s): Jul 27, 2023

Reporting Summary

Nature Portfolio wishes to improve the reproducibility of the work that we publish. This form provides structure for consistency and transparency in reporting. For further information on Nature Portfolio policies, see our Editorial Policies and the Editorial Policy Checklist.

Statistics

For all statistical analyses, confirm that the following items are present in the figure legend, table legend, main text, or Methods section.

n/a	Confirmed	
☐	☒	The exact sample size (*n*) for each experimental group/condition, given as a discrete number and unit of measurement
☐	☒	A statement on whether measurements were taken from distinct samples or whether the same sample was measured repeatedly
☐	☒	The statistical test(s) used AND whether they are one- or two-sided<br>*Only common tests should be described solely by name; describe more complex techniques in the Methods section.*
☐	☒	A description of all covariates tested
☐	☒	A description of any assumptions or corrections, such as tests of normality and adjustment for multiple comparisons
☐	☒	A full description of the statistical parameters including central tendency (e.g. means) or other basic estimates (e.g. regression coefficient) AND variation (e.g. standard deviation) or associated estimates of uncertainty (e.g. confidence intervals)
☐	☒	For null hypothesis testing, the test statistic (e.g. *F*, *t*, *r*) with confidence intervals, effect sizes, degrees of freedom and *P* value noted<br>*Give P values as exact values whenever suitable.*
☒	☐	For Bayesian analysis, information on the choice of priors and Markov chain Monte Carlo settings
☒	☐	For hierarchical and complex designs, identification of the appropriate level for tests and full reporting of outcomes
☒	☐	Estimates of effect sizes (e.g. Cohen's *d*, Pearson's *r*), indicating how they were calculated

Our web collection on statistics for biologists contains articles on many of the points above.

Software and code

Policy information about availability of computer code

Data collection	Flow cytometry data was acquired using BD FACSDiva Software version 9.0.1 (Beckton Dickinson), BD FACSuite RUO v.1.5, MATLAB The MathWorks Inc. (2022), MATALB version: 9.12.0 (R2022a).
Data analysis	Flow cytometry data was analyzed using GraphPad PRISM version 6.07, FlowJo version 10.6.1 (Miltenyi Biotec), BD FACSuite RUO v.1.5, The MathWorks Inc. (2022), MATALB version: 9.12.0 (R2022a), and Python (version 3.7.9).

For manuscripts utilizing custom algorithms or software that are central to the research but not yet described in published literature, software must be made available to editors and reviewers. We strongly encourage code deposition in a community repository (e.g. GitHub). See the Nature Portfolio guidelines for submitting code & software for further information.

Data

Policy information about availability of data

All manuscripts must include a data availability statement. This statement should provide the following information, where applicable:

- Accession codes, unique identifiers, or web links for publicly available datasets
- A description of any restrictions on data availability
- For clinical datasets or third party data, please ensure that the statement adheres to our policy

The source data to regenerate all figures and table in this study is provided in a Source Data file.

Research involving human participants, their data, or biological material

Policy information about studies with human participants or human data. See also policy information about sex, gender (identity/presentation), and sexual orientation and race, ethnicity and racism.

Reporting on sex and gender	N/A
Reporting on race, ethnicity, or other socially relevant groupings	N/A
Population characteristics	Included in Table 1 and S1.
Recruitment	Described in the "Participants and treatment" Results section.
Ethics oversight	Described in the "Participants and treatment" Results section.

Note that full information on the approval of the study protocol must also be provided in the manuscript.

Field-specific reporting

Please select the one below that is the best fit for your research. If you are not sure, read the appropriate sections before making your selection.

☒ Life sciences ☐ Behavioural & social sciences ☐ Ecological, evolutionary & environmental sciences

For a reference copy of the document with all sections, see nature.com/documents/nr-reporting-summary-flat.pdf

Life sciences study design

All studies must disclose on these points even when the disclosure is negative.

Sample size	Sample size was determined by the sample size of a previous clinical trial (NCT 03282656).
Data exclusions	No data was excluded.
Replication	Results were replicated by repeated measurements in the same subjects at different times. Assays stability was confirmed by repeated measurements in the same specimen. Reproducibility over all the main switch results was verified by three different assays with three different measurement principles.
Randomization	Subjects were grouped by BCL11A and HU treatment. BCL11A subjects correspond to all subjects included in the non-randomized clinical trial (NCT 03282656) and with at least 4 months of treatment. The HU subjects were randomly selected and were subdivided into two groups, HU High Responder and HU Low responder, solely on the basis of HbF%. HU High Responder cohort has HbF levels greater than 20% while HU Low Responder cohort has HbF levels equal or lower than 20%.
Blinding	Blinding was not possible because the previous clinical trial was an open-label and single arm cohort study. Blinding was not possible also because the specimen collection for the HU group started later.

Reporting for specific materials, systems and methods

We require information from authors about some types of materials, experimental systems and methods used in many studies. Here, indicate whether each material, system or method listed is relevant to your study. If you are not sure if a list item applies to your research, read the appropriate section before selecting a response.

Materials & experimental systems

n/a	Involved in the study	
☐	☒	Antibodies
☒	☐	Eukaryotic cell lines
☒	☐	Palaeontology and archaeology
☒	☐	Animals and other organisms
☐	☒	Clinical data
☒	☐	Dual use research of concern
☒	☐	Plants

Methods

n/a	Involved in the study	
☒	☐	ChIP-seq
☐	☒	Flow cytometry
☒	☐	MRI-based neuroimaging

Antibodies

Antibodies used	HbF-mass assay and HbF/cell and HbS/cell assay: Mouse monoclonal antibodies (Ab) against Human gamma-globin chain (IQ Products: IQP-363-INT-4) or Human betaS-globin chain (Rockland: 200-301-GS5) were used in this study. Anti-gamma-globin chain Ab was coupled to phycoerythrin (R-PE) (by IQ Products) and anti-betaS-globin chain Ab was coupled to pacific blue (PB) using an Ab conjugation kit (Thermofisher).<br><br>F-cell assay: mouse anti Human Fetal Hemoglobin R-PE (ThermoFisher: MHFH04, clone HBF-1)
Validation	HbF-mass assay and HbF/cell and HbS/cell assay: Mouse monoclonal Ab against Human gamma-globin chain (IQ Products: IQP-363-INT-4, clone WBAC HbF1) was validated in PMID: 32681733 and in the Extended data Figure 4.<br>Mouse monoclonal Ab against Human betaS-globin chain (Rockland: 200-301-GS5, clone 23E5.H6.G6.C1.H7.F7.G9.F6) was validated in PMID: 35075288 and in the Extended data Figure 4.<br><br>F-cell assay: Mouse anti Human Fetal Hemoglobin R-PE (ThermoFisher: MHFH04) meets the requirements of the Life Technologies Quality System at the time of release.

Clinical data

Policy information about clinical studies
All manuscripts should comply with the ICMJE guidelines for publication of clinical research and a completed CONSORT checklist must be included with all submissions.

Clinical trial registration	NCT 03282656
Study protocol	NCT 03282656
Data collection	NCT 03282656 and comparator group data was collected at Boston Children's Hospital from 2020 to 2022.
Outcomes	For the comparator group, only laboratory endpoints were assessed.<br><br>For the BCL11A subjects the primary and secondary endpoints we defined by the clinical trial NCT 03282656:<br>-Primary outcome measures were defined as the rescue of hematopoiesis after conditioning (defined by absolute neutrophil count (ANC) greater than or equal to 0.5 x 109 /L for three consecutive days), achieved within 7 weeks following infusion (i.e., "primary engraftment").<br>-Secondary outcome measures we defined as the presence of the transgene (vector copy number) in the following samples: i) Peripheral blood cells [measured in whole blood, mononuclear cells, and the following sorted populations: CD3+, CD15+, CD19+, and CD56+] at 6 weeks, and then every 6 months until 2 years after gene transfer.

Flow Cytometry

Plots

Confirm that:

☒ The axis labels state the marker and fluorochrome used (e.g. CD4-FITC).

☒ The axis scales are clearly visible. Include numbers along axes only for bottom left plot of group (a 'group' is an analysis of identical markers).

☒ All plots are contour plots with outliers or pseudocolor plots.

☒ A numerical value for number of cells or percentage (with statistics) is provided.

Methodology

Sample preparation	HbF-mass assay and HbF/cell and HbS/cell assay: Intracellular hemoglobin assessment by flow cytometry was performed as described in PMID: 32681733 and in PMID: 35075288. Every analysis was performed on glycerol-frozen red blood cells that were thawed, fixed and permeabilized before staining with Ab. Cells were incubated for 15 minutes, shielded from light at room temperature, then washed with PBS and analyzed on a flow cytometer immediately. The mouse anti-human fetal hemoglobin is a commercialy available monoclonal antibody.<br><br>F-cell assay: red blood cells were fixed, permeabelized and stained with anti-HbF (R-PE) Ab as described in PMID: PMC7962145. Post incubation the samples were acquired using the BD FACSLyric instrument. F-cell reports were generated using the BD FACSuite RUO v.1.5.
Instrument	HbF-mass assay and HbF/cell and HbS/cell assay: BD FACSCanto II system 8-color flow cytometer (BD Biosciences)<br><br>F-cell assay: BD FACSLyric (BD Biosciences) 3L12C Instrument RUO (BD Biosciences)
Software	HbF-mass assay and HbF/cell and HbS/cell assay: Cells were acquired using the BD FACSDiva Software version 9.0.1 and data was analyzed using FlowJo version 10.6.1

	F-cell assay: Cells were acquired using the BD FACSuite RUO v.1.5 and data was analyzed using FlowJo version 10.8.1
Cell population abundance	HbF-mass assay and HbF/cell and HbS/cell assay: A minimum of 100,000 RBCs were recorded per sample analyzed (with a purity >99%).<br><br>F-cell assay: Approximatively 125,000 fixed and permeabelized RBCs were stained and acquired. A minimum of 10,000 RBCs events were recorded for each sample.
Gating strategy	HbF-mass assay and HbF/cell and HbS/cell assay: Red blood cells were first gated on forward scatter area (FCS-A) versus side scatter area (SSC-A) plot. Doublet exclusion was done by selecting single cells on FSC-H (FSC-Height) plotted against FSC-W (FSC-Width) and then on SSC-H versus SSC-W. In case of HbF quantification in only HbS-positive cells, the latter population was gated on HbS-PB-A plooted against HbF-PE-A.<br><br>F-cell assay: RBCs were gated on FCS-A vs SSC-A plot, followed by doublets exclusion as described above. A positive cutoff point was set at 0.1% above the negative population of unstained control sample.

☒ Tick this box to confirm that a figure exemplifying the gating strategy is provided in the Supplementary Information.
